# Assessment of Vancomycin MIC Creep Phenomenon in Methicillin-Resistant *Staphylococcus aureus* isolates in a Tertiary Care Hospital of Lahore

**DOI:** 10.12669/pjms.36.7.3273

**Published:** 2020

**Authors:** Faiqa Arshad, Sidrah Saleem, Shah Jahan, Romeeza Tahir

**Affiliations:** 1 Dr. Faiqa Arshad Ph.D. Scholar. Department of Microbiology, University of Health Sciences, Lahore, Pakistan; 2 Dr. Sidrah Saleem, MBBS, M.Phil., PhD (Microbiology), Professor & Head, Department of Microbiology, University of Health Sciences, Lahore, Pakistan; 3 Dr. Shah Jahan, PhD (Molecular Biology), Associate Professor, Department of Immunology, University of Health Sciences, Lahore, Pakistan; 4 Dr. Romeeza Tahir, M.Sc., M.Phil., PhD (Immunology) Assistant Professor, Department of Immunology, University of Health Sciences, Lahore, Pakistan

**Keywords:** Methicillin-resistant *Staphylococcus aureus* (MRSA), Vancomycin intermediate *Staphylococcus aureus* (VISA), Vancomycin-resistant *Staphylococcus aureus* (VRSA), Minimum inhibitory concentration (MIC)

## Abstract

**Objective:**

To assess vancomycin MIC creep phenomenon in methicillin-resistant *Staphylococcus aureus* isolated from clinical specimens.

**Methods:**

This descriptive study was conducted in Microbiology department of University of Health Sciences, Lahore from January 2016- December 2019. In this study, vancomycin MICs were revealed by E test method for clinical MRSA strains. For the final evaluation, a single isolate from each patient was taken. The reported vancomycin MICs results were used and the values were not rounded up to the next upward value. For every study year, MIC50, MIC90, median and geometrical mean MIC, percentages of susceptible and resistant strains were calculated.

**Results:**

A total of 352 MRSA strains were isolated out of 2704 staphylococcal isolates. Our study showed elevated vancomycin MIC among MRSA isolates. The majority of isolates showed MIC values ≥1.5µg/ml. MIC50, MIC 90 was constant throughout four years period. However, geometric mean MIC increased gradually during the study period. The MIC greater than base year median was overall 17.3%. A complete shift can be observed between MIC “1.0” and “2.0” the percent of cases with MIC “1.0” decreased and with MIC “2.0” increased over time crossing each other in 2017.

**Conclusion:**

Vancomycin MIC creep was identified in clinical isolates of MRSA, during four years of study period. Even though there is an absence of VISA and VRSA strains; this significant increase in vancomycin MIC trend is indeed worrying for the clinicians about the threat of potential failure of treatment in MRSA infections.

## INTRODUCTION

MRSA is a superbug and is a serious human health issue that is considered as one of the foremost causes of healthcare and community acquired infections.^1^ MRSA is unaffected by methicillin and many lactam antibiotics, such as oxacillin, cefoxitin, cephalosporins and carbapenems.^2^ The strains of MRSA have been now endemic in a large number of hospitals throughout the world and predominantly affect patients, who have undergone major surgical procedures and those who are in intensive care units.^3^

Vancomycin has remained a mainstay of treatment for serious MRSA infections for many years.^4^ However, an increase in vancomycin MIC values in MRSA isolates is a serious concern because it has been indicated in many studies that the patients with high vancomycin MIC values even within the sensitive limit are ending with treatment failure.^5^ This rise in vancomycin MIC standards over the period is called “MIC creep”. The term “creep” may be classified as a “gradual and unnoticed movement or shift.” Thus, vancomycin MIC creep should be known as a steady rise in the central disposition of the vancomycin MIC levels. It results from the long term and excessive use of vancomycin under sub-optimal doses.^6^

Creep phenomenon is globally reported to result in therapeutic failure, increase morbidity and increase relapse rate, slow clinical response and higher relapse rate.^7^ Evidently, creep is a regional problem consequently, local evaluation of susceptibility profiles is important for the clinical management of local MRSA infection.^8^ Now, there is a need to assess the existence of creep trend and warn the clinicians of these disastrous strains.^9^ Suitable analytical patterns for evaluation of the correlation between vancomycin MICs and scientific consequence of MRSA infections need to be investigated.^10^ Therefore, there is a dire need to identify the trend of vancomycin MICs in our local area.

The present research was designed to assess MIC creep for clinical isolates of MRSA against vancomycin over a 4-year period in a tertiary care institute, Lahore.

## METHODS

The study was carried out after Ethics Committee/IRB approval with number UHS/Education/126-18/3731, dated 23-11-2018. In this study, MRSA strains were obtained from different clinical specimens of hospitalized subjects with MRSA infection recognized from medical history and clinical microbiology laboratory, from 2016-2019 at Lahore General Hospital Lahore. The samples included blood, pus, wound swab, respiratory tract (tracheal aspirate and bronchoalveolar lavage) CSF, synovial fluid, urine and sputum. Only one sample per patient was incorporated into the study. Only the first isolate was tested in case there were more than one sample from the same patient.

Over-all 352 isolates were recovered from 2704 *S.aureus* isolates from various clinical samples of in-door patients. The specimens processed in the microbiology lab of the University of Health Sciences, Lahore were inoculated on blood agar plates and incubation was done at 35-37°C aerobically for 24hours. Following standard microbiological techniques; primary detection of *S. aureus* was carried out by observing the colony morphology on blood agar plates, Gram staining and catalase. Biochemical tests like coagulase and DNase were done for organism confirmation. The phenotypic screening for methicillin resistance was determined by the modified Kirby-Bauer disc diffusion method using 30µg cefoxitin disc (Oxoid) according to CLSI guiding principles.^11^ A bacterial suspension was adjusted for each strain, according to 0.5 McFarland turbidity standards and inoculation was done on Mueller Hinton agar (MHA). Zone of inhibition was determined after overnight incubation at 35^o^C. The results were interpreted according to CLSI standards 2019. MRSA ATCC 33591 and methicillin-susceptible *S. aureus* (MSSA) ATCC 25923 were used as Controls in parallel while performing all phenotypic and genotypic test runs.^11^

MICs of vancomycin were detected by E-strips. The inoculum was prepared according to 0.5 McFarland turbidity standards (10^6^cfu/ml). Using a sterilized cotton swab, isolates were inoculated on to Muller Hinton agar. E-strips of vancomycin were applied to it. Incubation was done with controls for 18-24 hours at 37°C aerobically. MIC results were interpreted as susceptible or resistance according to criteria set by CLSI. For vancomycin, MICs ≤2µg/mL were taken into account as sensitive, 4-8 µg/mL intermediate and ≥16 µg/mL were considered in resistant zone 4,11

Statistical analysis for assessment of MIC creep was done by determining different parameters like MIC50, MIC90 (median, 90^th^ percentile), mode, geometrical mean MIC, susceptible and resistant percentages for vancomycin in each year. All the determined susceptibility markers were assessed in each year and plotted over time to assess for vancomycin MIC trends.

Likelihood ratio test was applied to see if the distribution of isolate with MIC≥1.50 µg/mL for vancomycin differed over the years. p-values were reported accurate to three decimal places.

## RESULTS

**Fig.1:**
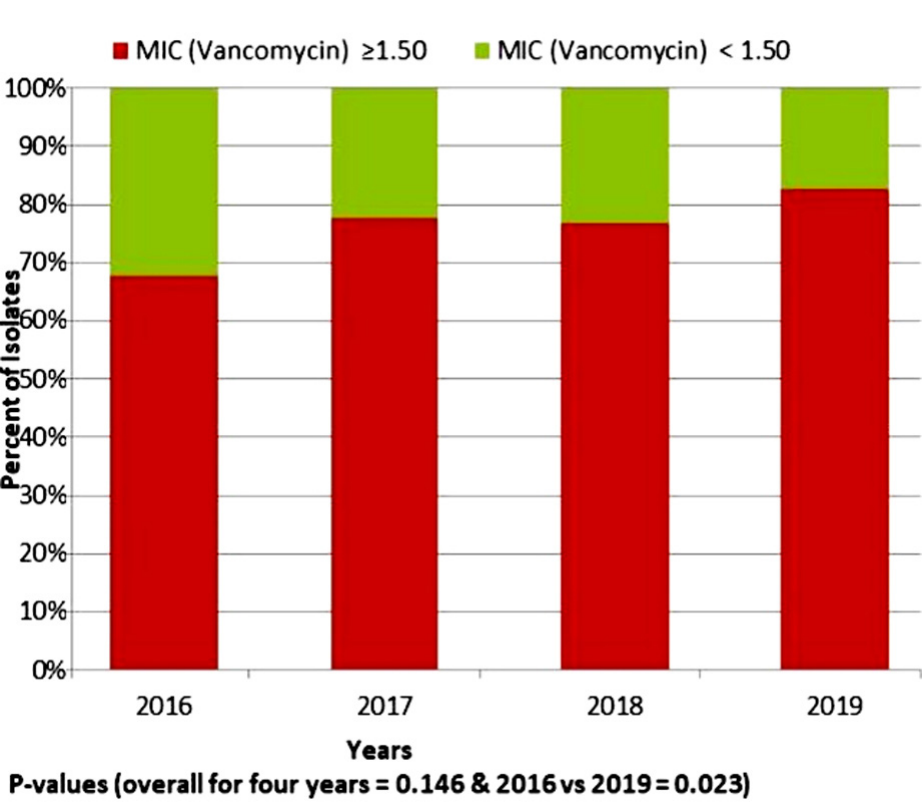
The distribution and trend of vancomycin for clinical isolates of methicillin-resistant Staphylococcus aureus for years 2016-2019.

During this study project, over-all 352 MRSA isolates were recovered from 2704 isolates of *S. aureus*. All MRSA strains were susceptible to vancomycin. MIC for vancomycin remained on rise continuously and by the end of study at year 2019, 82.6% cases were with MIC ≥1.5 as compared to 67.8% in 2016. This difference for overall comparison among four years had a p-value 0.146, when compared only between 2016 and 2019 the cases with MIC ≥ 1.5 µg/mL were significantly higher with p-value 0.023. (Fig.1).

Specimen wise distribution of MRSA isolates was observed as well. It was noticed that there was no significant difference for specimen type over the years with p-value = 0.998. The distribution pattern was not different. Pus, blood and wound swab were the three most common specimens for these isolates (Table-I).

**Table-I:** The sources of the clinical methicillin-resistant *Staphylococcus aureus* (MRSA) isolates (2016-2019).

Specimen	Year								Total	

	2016		2017		2018		2019	

	n	%	n	%	n	%	n	%	n	%
Pus	43	51.2	45	50.0	39	45.3	48	52.2	175	49.7
Blood	13	15.5	15	16.7	12	14.0	16	17.4	56	15.9
Wound swab	11	13.1	12	13.3	15	17.4	13	14.1	51	14.5
Fluids & Aspirates	5	6.0	8	8.9	6	7.0	5	5.4	24	6.8
CSF	4	4.8	3	3.3	5	5.8	3	3.3	15	4.3
Sputum	3	3.6	2	2.2	4	4.7	2	2.2	11	3.1
CVP tip	2	2.4	2	2.2	3	3.5	4	4.3	11	3.1
Urine	3	3.6	3	3.3	2	2.3	1	1.1	9	2.6

Total	84	100.2	90	100.0	86	100.0	92	100.0	352	100.0

Likelihood ratio = 6.90, P-value = 0.998

For vancomycin, the MIC was also recorded for each case. It was noted that the median MIC, throughout all the years, was 1.5 µg/mL and the 90^th^ percentile was 2.0 µg/mL. The geometric mean measured in 2016 was 1.3 µg/mL, which increased gradually till 2019 to 1.5 µg/mL. In 2016 and 2017 the maximum MIC was 2.0 µg/mL for all years. As compared to 2016, 9.9% cases increased with MIC greater than 1.5 in 2017. This increment for the year 2018 was 8.8%, and that for 2019 was 14.7%. So in three years, MICs greater than base year median was overall 14.7 % (Table-II).

**Table-II:** Statistics and susceptibility regarding vancomycin MICs (µg/mL) for clinical MRSA strains (2016-2019).

Year	No of strains	MIC50 (µg/mL)	MIC90 (µg/mL)	MIC <1.5 µg/mL	MIC≥ 1.5 µg/mL	Geometric mean MIC	Modal MIC	MIC range	Percent of isolates MIC > baseline year ^a^Median MIC	%S/%R

				n (%)	n (%)	µg/mL				
2016	84	1.50	2.00	27 (32.1)	57 (67.9)	1.3	1.50	(0.5 - 2.0)	--	100/0
2017	90	1.50	2.00	20 (22.2)	70 (77.8)	1.4	1.50	(1.0 - 2.0)	9.9	100/0
2018	86	1.50	2.00	20 (23.3)	66 (76.7)	1.4	1.50	(0.75 - 2.0)	8.8	100/0
2019	92	1.50	2.00	16 (17.4)	76 (82.6)	1.5	1.50	(1.0 - 2.0)	14.7	100/0

Total	352	1.50	2.00	83(23.6)	269 (76.4)	1.4	1.50	(0.5 - 2.0)	8.5	100/0

**Fig.2:**
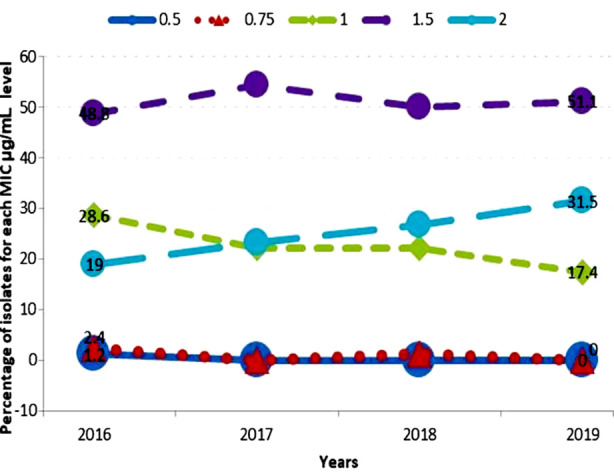
Vancomycin MIC population distribution 2016-2019.

It can be seen clearly in Fig.2 that 0.5 and 0.75 MIC was very rare from 2016 to 2019, almost touching zero. The MIC “1.0” had a clear decline from 2016 to 2019, starting from 28.6% of cases to 17.4% in 2019. Conversely, the cases with MIC “2.0” increased during five years from 19.0% to 31.5%. The maximum number of cases had MIC 1.5 through all the years. These were 48.8% in 2016, 54.4% in 2017, 50.0% in 2018 and then finally 51.1% in 2019. A complete shift can be observed between MIC “1.0” and “2.0” the percent of cases with MIC “1.0” decreased and with MIC “2.0” increased over time crossing each other in 2017.

## DISCUSSION

MRSA has been globally proved to be serious threat to public health. Vancomycin is one of the mainstays for the cure of MRSA infections. However, reduced susceptibility of vancomycin for MRSA infections has been a serious concern over the past few decades.^12^

Our study showed elevated vancomycin MIC for MRSA for four years from 2016-2019 in a large tertiary care institute of Pakistan. Even though there was an absence of VISA and VRSA strains in our study, still a significant shifting trend towards higher MIC values raises a serious concern regarding potential therapeutic failure by adversely effecting vancomycin activity against MRSA as indicated in a study done in India.^13^ A shift towards higher vancomycin MIC values have been documented in studies conducted in different areas worldwide. A similar study conducted in Malaysia discussed that rise in vancomycin MIC value in a time period even being in the susceptible range is labelled as “creep phenomenon”. The study mentioned that creep phenomenon cannot be recognized on small scale studies because the difference noted may be very minute and are within susceptible range but has serious clinical impacts in future. To recognize this shift in MIC values, we need a study over a period of few years. Majority of isolates in our study had MIC values ≥1.5µg/mL.^14^ A study done by Cheema et al revealed that 71% isolates had vancomycin MIC of 2 µg/mL while 29 % isolates had MIC values of 1 µg/mL.^15^ A study conducted by Ejaz et al found that all of the MRSA isolate were sensitive to vancomycin.^16^ However 4% of the *S. aureus* strains were reported as resistant to vancomycin in a study done in Allied Hospital Faisalabad, Pakistan.^17^ Lower vancomycin MIC values to the strains of MRSA ranged from 0.125 μg/mL to 1 μg/mL were observed in a study conducted in Nepal.^18^ In our study, 0.5 and 0.75 MIC was very rare throughout 2016 to 2019 as indicated in another study.^12^ In contrast, a study reported that the occurrence of isolates for which the vancomycin MIC was >1 µg/mL was very uncommon, with no increased trend. They tried to find out the possibility of vancomycin MIC creep against MRSA in a large multicenter study conducted in different states of U.S.^19^

Regarding the specimen wise distribution of MRSA isolates, it was found out that pattern was not different by each passing year. Most of the isolates were recovered from samples of pus, blood and wound swabs. Similar findings have been observed in the previous study.^20^ Most of the studies identified the creep phenomenon by using the E test method of susceptibility testing as done in this study.^12^ E test method is more reliable, sensitive and can accurately detect small MIC changings in vancomycin.^8^

In this study, MIC50, MIC 90 was constant throughout four years period. However, geometric mean MIC raised gradually during the study duration. The MIC greater than base year median was overall 17.3%. Another study reported that geometric mean MIC is a more sensitive marker and reflects the changes in MIC distribution more precisely than traditional susceptibility markers of MIC 50, MIC 90, MIC range and percent susceptibility and percent resistant.^21^ Most studies in Pakistan are not designed to assess more subtle changes in MIC distribution over time. They used traditional markers over a specific period that can disguise tendencies that occur in a given setting over a period of few years.^20^

Studies in different parts of the world have reported vancomycin susceptibility changes over time in single-center and for a similar period of time, as in this study.^22^,^23^ Yeh et al did study in Taiwan for five years and found an upward trend of vancomycin MIC than baseline year.^22^ Evaluation for creep for three years in a hospital of Portugal reported that creep did not exist in their institution. They suggested that this phenomenon seems not generalized, so each institution should monitor vancomycin MIC values autonomously.^6^ A study was done in two cities of Germany on blood culture isolates by E test method. They demonstrated that creep exists in City A based on a substantial increase in the number of isolates with a MIC of 1 µg/mL or higher, but there was no change in City B. They also suggested that health-care settings should observe their own local status of vancomycin MIC values for MRSA strains.^8^

The clinical importance of the increasing trend of vancomycin MIC values, even being in the susceptible range, i.e., creep phenomenon, has been mentioned in various studies. Studies warn that even a minute increase in MIC below the breakpoint is resulting in therapeutic failure when given to patients and is a major medical concern.^24^ A study conducted in Barcelona, Spain, stated that there is a greater probability of therapeutic failure if the strains had a MIC ≥1.5 µg/mL. Moreover, the death rate related to MRSA bacteremia was significantly higher when vancomycin was empirically used for medication of infectious strains showing high vancomycin MIC values.^25^

### Limitations of the study

It is single center study that observed vancomycin MIC creep phenomenon in MRSA isolates of a Tertiary care Hospital of Lahore. We recommend that more studies should be conducted in different hospital of Pakistan to monitor the local Vancomycin MICs Trends over a period of time.

## CONCLUSION

Vancomycin MIC creep was found among clinical MRSA isolates during four years of the study period. Even though there is an absence of VISA and VRSA strains, a significant increase in vancomycin MIC trend within the susceptible range is indeed worrying for the clinicians about the threat of potential therapeutic failure of MRSA infections. Moreover, such studies should be continued in the future in Pakistani hospitals so that we can timely detect the vancomycin-intermediate and resistant strains because the increasing trend of vancomycin can lead the first step to VISA. Moreover, clinical laboratories should implement meticulous techniques for the determination of vancomycin with accurate precision.

### Authors’ Contribution:

**FA:** Entire Research work (conception & design, Data analysis& interpretation). Responsible and accountable for the accuracy or integrity of the work.

**SS:** Conceptualization & Overall supervision of research work, Final approval of version to be submitted. Responsible and accountable for the accuracy or integrity of the work.

**SJ, RT:** Supervision of research work, Revising critically for important intellectual content.
